# Nanomaterial-Based Zinc Ion Interference Therapy to Combat Bacterial Infections

**DOI:** 10.3389/fimmu.2022.899992

**Published:** 2022-06-30

**Authors:** Yongbin Wei, Jiaming Wang, Sixuan Wu, Ruixue Zhou, Kaixiang Zhang, Zhenzhong Zhang, Junjie Liu, Shangshang Qin, Jinjin Shi

**Affiliations:** ^1^ School of Pharmaceutical Sciences, Zhengzhou University, Zhengzhou, China; ^2^ Key Laboratory of Targeting Therapy and Diagnosis for Critical Diseases, Zhengzhou University, Zhengzhou, China; ^3^ Key Laboratory of Key Drug Preparation Technology Ministry of Education, Zhengzhou University, Zhengzhou, China

**Keywords:** antibacterial therapeutics, nanomaterials, zinc ion interference, targeted delivery technology, zinc homeostasis

## Abstract

Pathogenic bacterial infections are the second highest cause of death worldwide and bring severe challenges to public healthcare. Antibiotic resistance makes it urgent to explore new antibacterial therapy. As an essential metal element in both humans and bacteria, zinc ions have various physiological and biochemical functions. They can stabilize the folded conformation of metalloproteins and participate in critical biochemical reactions, including DNA replication, transcription, translation, and signal transduction. Therefore, zinc deficiency would impair bacterial activity and inhibit the growth of bacteria. Interestingly, excess zinc ions also could cause oxidative stress to damage DNA, proteins, and lipids by inhibiting the function of respiratory enzymes to promote the formation of free radicals. Such dual characteristics endow zinc ions with unparalleled advantages in the direction of antibacterial therapy. Based on the fascinating features of zinc ions, nanomaterial-based zinc ion interference therapy emerges relying on the outstanding benefits of nanomaterials. Zinc ion interference therapy is divided into two classes: zinc overloading and zinc deprivation. In this review, we summarized the recent innovative zinc ion interference strategy for the treatment of bacterial infections and focused on analyzing the antibacterial mechanism of zinc overloading and zinc deprivation. Finally, we discuss the current limitations of zinc ion interference antibacterial therapy and put forward problems of clinical translation for zinc ion interference antibacterial therapy.

## Introduction

Bacterial infections, especially multidrug-resistant (MDR) bacteria, are considered a major threat to global healthcare due to the emergence and rapid spread of antibiotic resistance (1–3). According to the WHO in 2019, antimicrobial resistance (AMR) had been declared a top 10 global health threat. It is predicted that around 10 million annual deaths are expected by 2050 if the situation is not improved (4). Infection by these bacteria could cause many types of serious diseases such as pneumonia and sepsis. The bacteria could invade the human with the help of the motility of bacterial flagella and trigger a severe cytokine-mediated immune response (5–7). This severe immune response could damage the host’s normal organs, causing organ dysfunction (8). Moreover, some important virulence factors released by bacteria could also cause a series of toxicities (9, 10). With the aggravation of local bacterial infection, systemic sepsis is developed, which is defined as the host inflammatory response to severe, life-threatening infection (11–13). During sepsis, the release of pathogen-associated molecular patterns (PAMPs) and damage-associated molecular patterns (DAMPs) perpetuates systemic inflammation, a collapse of cardiovascular function, multiple organ dysfunction, and septic shock. Despite advanced supportive care, the mortality rates in severe sepsis and septic shock reach up to 40% (14, 15). Currently, the clinical treatment options for MDR bacteria are extremely limited, and it is difficult to develop novel antibiotics. Therefore, it is urgently needed to treat the infection caused by this bacterium.

Many metal ions (16, 17) have been reported to possess a broad spectrum of antibacterial activity. Silver and copper metals have been used as antibacterial agents for thousands of years. Due to the unique physical and chemical properties of nanomaterials, metal-based nanoparticles (NPs), such as silver NPs, and copper NPs, have emerged as promising options in the antibacterial field (16, 18, 19). Many studies have confirmed that the antibacterial effect of these metals silver and copper is mainly due to the ions produced (20). The antibacterial mechanisms of silver and copper ions are involved in reactive oxygen species (ROS) production, membrane damage, and destruction of ATP synthesis (21). In this framework, silver- and copper-based nano-antibacterial therapy has been widely studied by researchers and used in cosmetics, medical devices coating, and other fields (18). However, antibacterial therapies based on heavy metal NPs also have some limitations. I) Silver and copper NPs could induce cell membrane perforation, ROS damage, and liver dysfunction (18). II) These heavy metal ions would accumulate in mammalian cells, causing serious toxicity that impairs the physiology of the normal cell. III) Recently, some studies (22) have shown that bacteria are becoming resistant to silver ions, which means that the antibacterial potency of silver ions/silver NPs is dropping. For TiO_2_ NPs, it generally acts as a photocatalytic agent (23, 24) to enhance the antibacterial efficacy. The penetration depth of UV-light or near-IR (NIR) still severely limits the application of the TiO_2_-based nanosystems. Therefore, a new strategy needs to be developed to fight against bacteria.

**Figure 1:**
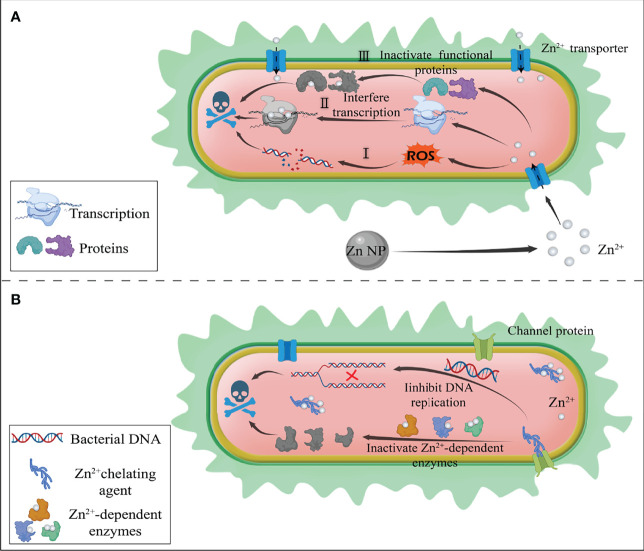
Schematic diagram of the antibacterial mechanism of zinc ion interference therapy. **(A)** Zinc ion overloading strategy. Zinc-loaded nanodelivery systems release large amounts of zinc ions at the site of bacterial infection. The excess zinc ions produce the following antibacterial effects: I) interaction with the thiol group of bacterial respiratory enzymes resulting in the production of intracellular ROS, thus damaging bacterial DNA: II) interference with bacterial transcriptional regulation; III) inactivation of functional proteins leading to metabolic dysfunction of bacteria. **(B)** Zinc ion deprivation strategy. Zinc chelating agents deprive intracellular zinc ions, resulting in the imbalance of zinc ion homeostasis. The lack of zinc ions could lead to the inactivation of Zn^2+^-dependent enzymes, affecting the structure of chromatin and inhibiting the replication of DNA, thus causing bacterial death.

Competition of transition metal ions between host and pathogen is a key battleground for infectious diseases (25). The result of the competition could determine whether the infection is successfully established. As one of the most important essential metals in the human body, Zn plays a key role in many signal transduction processes of organisms and acts as a key structural cofactor in the biological functions of many proteins (26–29). Firstly, Zn(II) is a cofactor of many bacterial proteins; for example, New Delhi metallo-β-lactamase 1 (NDM-1) (30), a β-lactamase with double Zn(II) as its active center, activates nucleophilic water molecule to destroy the β-lactam ring of antibiotics (31). Then, Zn(II) is related to bacterial transcriptional regulation and the maintenance of gene stability by different paths. It could also stabilize the structure of chromatin and affect the replication of DNA (32). Zinc-sensitive modulators are used to alter gene expression in response to changes in intracellular zinc (33). Zn(II) is also responsible for the virulence of bacteria by the zinc uptake regulator. For example, ZnuABC (a zinc uptake system) plays a critical role in facilitating bacterial adhesion to epithelial cells (25, 34). Therefore, bacterial intracellular zinc imbalance would result in the dysfunction of a range of functional proteins (35), organism metabolic disorders (36), and transcription imbalance (33). For example, small molecule inhibitors such as disodium ethylenediaminetetraacetate (EDTA) (37) and aspergillomarasmine A (AMA) (38) could chelate zinc ions in bacteria, resulting in the inactivation of NDM-1. Interestingly, excess Zn(II) naturally possesses superior antibacterial activity. Bacteria intracellular accumulation of Zn(II) would interact with the thiol group of bacterial respiratory enzymes and result in the inhibition of their function. This inactivation of respiratory enzymes causes a mismatch during the electron transfer process in the bacterial respiratory chain, resulting in the production of intracellular ROS (39, 40). The intracellular ROS would irreversibly induce bacterial DNA damage and induce bacterial membranes lipid peroxidation, leading to bacterial death (39). Moreover, compared to heavy ions/NPs, zinc is safer for normal cells. Zinc is a mineral element essential to humans (41) and exhibits strong antibacterial activity even when administered in small doses. Zinc-based nanomaterials are easy to decompose, so they could be better metabolized from the body. Those degradable nanomaterials radically reduce the inherent toxicity of nanomaterials. Moreover, zinc ions are versatile. They could not only exert an excellent antibacterial function but also act as nutrients or stimulus signals for damaged tissues. In wound and osteomyelitis mouse models, Zn(II) accelerates wound healing and improves osteogenic differentiation of bone marrow mesenchymal stem cells (42). Therefore, with the ever-developing MDR bacteria and the slow pace of development of efficient antibiotics, Zn(II) interference therapy might offer an alternative option to combat bacterial infection. Based on the dual characteristics of zinc ion, zinc ion interference therapy could be divided into two strategies: zinc overloading and zinc deprivation. For the first, the Zn(II) overloading strategy could increase locally zinc ion concentration and achieve excellent antibacterial effects (**Figure 1A**). The Zn(II) deprivation strategy is used to break the zinc homeostasis by chelating the bacterial microenvironment or intracellular Zn(II) to kill bacterial species (**Figure 1B**). 

In the past decades, nanoscience and nanotechnology have flourished with outstanding progress (43, 44). Various antibacterial nanomaterials, including liposomes (45, 46), carbon-based nanomaterials (47, 48), noble metal NPs (49, 50), semiconductor NPs (51), and polymeric nanomaterials (52), are being developed as alternative therapeutic options to combat against bacterial infections. These nanomaterials not only could exert a good antibacterial activity by themselves (53) but also are loaded with antibiotics (54) or antimicrobial peptides (55). After further modification (56, 57), they could achieve targeted drug delivery to exert a synergistic antibacterial effect. In the post-antibiotic era, nanomaterial-based antimicrobial therapy is a promising antibacterial strategy.

Recently, owing to zinc-based antibacterial materials with a large surface/volume ratio (58, 59), pH-responsive release nanostructure (60), and good biocompatibility, nanomaterial-based zinc ion interference therapy against bacterial infection has attracted much attention and is widely used in various fields (61–63). It could be designed as targeting, environmentally responsive antibacterial delivery systems. It could also serve as a platform for integrating multiple modes of antimicrobial action against pathogens. Here, we review the recent advances in antibacterial research of zinc ion interference therapy and focus on its main physical and chemical properties and various mechanisms of antibacterial action. Finally, we discuss the challenges and prospects of utilizing zinc ion interference therapy in practical antibacterial applications.

## Zn(II) Overloading Strategy

More and more studies have shown that the bactericidal mechanism of Zn(II) involves the inactivation of many functional proteins and the production of ROS (36). The excess Zn(II) will bind the active-site thiols to inhibit the activity of functional proteins (35, 39). Moreover, the excess Zn(II) induces the production of large amounts of ROS in bacterial cells *via* interacting with the thiol group of bacterial respiratory enzymes (39). The ROS mediated by Zn(II) could damage cellular macromolecules and contribute to protein oxidation, lipid peroxidation, and DNA damage (64). Considering the excellent antibacterial property of Zn(II), the Zn(II) overloading strategy by delivering local excess of Zn(II) to fight against bacterial infections has attracted increasing attention from researchers, and numerous Zn(II)-based nanodelivery systems were developed to combat bacterial infections (65, 66). However, certain challenges still limit their application and therapeutic efficacy. Therefore, in the following, we will discuss the limitations of the Zn(II) overloading strategy and how to overcome the challenges.

### ZIF-8

**Figure 2:**
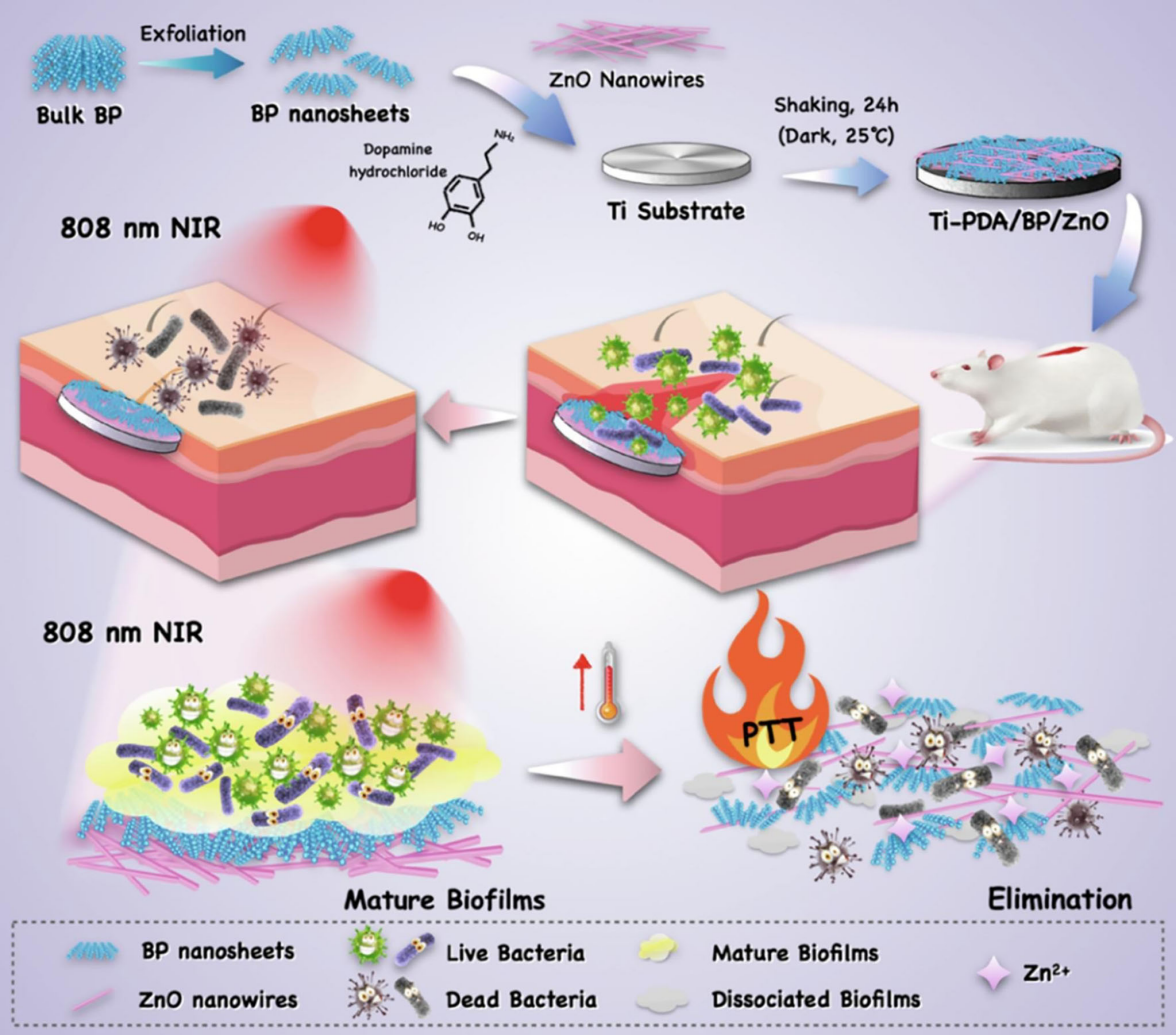
Schematic illustration of Mn-ZIF-8 nanocomposite for antibacterial effects on infected wound model by eliminating bacteria (79). Copyright 2021, WILEY.

Metal-organic framework (MOF) materials are a kind of coordination polymers developed rapidly in the past two decades. They are crystalline porous materials with periodic network structures formed by the self-assembly of transition metal ions and organic ligands (67, 68). They have high porosity, low density, large specific surface area, regular pore size, and diversified topological structure. They are a new type of porous material and have been widely used in catalysis (69, 70), energy storage (71), and biomedicine (72). Among the MOFs, the zinc-dimethylimidazole MOF-8 (ZIF-8) is made up of Zn(II) and dimethylimidazole and is one of the most widely studied MOFs. Given the simple synthesis method (72), high porosity (73), and pH-triggered structural collapse (60, 74), ZIF-8 has been widely used as a pH-responsive nanodelivery system for antibacterial applications, as shown in **Table 1**. Meanwhile, ZIF-8 could absorb a great deal of Zn(II) in the cavities and be an ideal candidate for Zn(II)-overloading therapy. Wu et al. (78) reported a biodegradable BSA@ZIF-8 for simultaneously ablating tumors and inhibiting infection. As a Zn(II) reservoir, BSA@ZIF-8 could release plenty of Zn(II) for antibacterial therapy. The excess Zn(II) could disrupt the metabolic activity of bacteria. Moreover, a Mn-doped ZIF-8 (79) was prepared to simultaneously achieve a favorable antibacterial effect and regulation of inflammatory response (**Figure 2**). Therefore, all of the above articles demonstrate the excellent antibacterial efficiency attributed to Zn(II) overloading therapy.

**Table 1:** Application of antibacterial ZIF-8-based nanodelivery system.

	Structural organization	Compositionand modifications	Antibacterial mechanism andZn release kinetics	Bacterial Species	Size	Method	Test value	Ref
pH-responsive	Pd(H)@ZIF-8@AP	Palladium nanoparticles loaded with hydrogen are encapsulated inside ZIF-8, and ascorbate palmitate (AP) was coated on the Pd(H)@ZIF-8	In situ pH-responsive hydrogen and Zn(II) release system could effectively kill bacteria and regulate inflammation response	*H. Pylori*	190 nm	One-pot process	>90%	(75)
	tetracycline (Tet)@ZIF-8@hyaluronic acid (HA) (TZH)	Antibiotics were encapsulated inside the ZIF-8. HA was added to functionalize the surface of the ZIF-8	Zn(II)/antibiotic synergistic system for the targeted highly efficient elimination of intracellular bacteria. The pH-response behaviors cause the release of Zn(II)	*S. aureus* and *Salmonella*	500 nm	One-pot water phase approach	MIC=0.5 μg/mL MIC=1.0 μg/mL	(76)
	MPDA@ZIF-8/PES	A ZIF-8-coated mesoporous polydopamine (MPDA) NPs and then loaded the heat-shock protein inhibitor-PES was prepared	The controlled release of PES and Zn(II) were triggered in the acidic environment of bacterial biofilm	*S. aureus*	150 nm	One-pot procedure	97.40%	(77)
	BSA@ZIF-8	BSA was coated on the surface of ZIF-8	BSA@ZIF-8 simultaneously inhibits the growth of bacteria treat tumors, which presents excellent biodegradability	MRSA and *E. coli*	153.9 nm	One-pot method	MIC=12.5μg/mL MIC=25μg/mL	(78)
	Mn-ZIF-8	Uniformly doping Mn^2+^/Mn^4+^ into the skeleton of ZIF-8	The enzymatic activity of Mn(II) and superiority of pore structure of ZIF-8 are effectively combined to realize bacteria-killing and inflammation modulation	*S. aureus* and *E. coli*	75 nm	One-pot method	99%	(79)
	ZIF-8-ICG@MNs	The photosensitizer indocyanine green (ICG) was loaded into the ZIF-8	pH-sensitive Zn(II) release nanoplatform amplified chemo-photodynamic treatment	*P. acnes*	110 nm	One-pot synthesis	≈100%	(80)
	ZIF-8@Levo/ LBL	Levofloxacin (Levo)-loaded ZIF-8 was deposition on the Ti implants. Then, the gelatin (Gel) and chitosan (Chi) multilayers are coated on Ti	The coating would reduce the hydrolysis of ZIF-8@Levo for a sustained release of Zn(II)	*E. coli* and *S. aureus*	189±35 nm	One-pot method	88.5% 86.4%	(81)
Light-triggered	QDs@ZIF-8	Colloidal semiconductor QDs were encapsulated into ZIF-8	Enhanced photocatalysis of ZIF-8 leads to the generation of Zn(II) and ROS	*S. aureus* and *E. coli*	151.72±25.06 nm	One-pot method	100%	(82)
	ICG@ZIF-8/PDA/Ag	The complex consists of encapsulated ICG, in situ reductions to generate Ag nanoparticles and the coating polydopamine	Chemo-photothermal synergistic therapy increased the antibacterial. The Zn(II) release was triggered by 808 nm laser irradiation	*E. coli* and *S. aureus*	480±16 nm	One-pot reaction	MBC=6.25μg/mL	(83)
	ZIF-8	ZIF-8	Photocatalysis is mediated by the production of Zn(II) and reactive oxygen species (ROS)	*E. coli*	95 nm	One-pot reaction	99.99%	(84)
	ZnO-CNP-TRGL	ZIF-8-derived nanocarbons	NIR-responsive ZIF-8 generates localized massive heat and abundant Zn(II)	*S. aureus*	50 nm	Carbonize oxidize and in situ polymerization	≈100%	(85)
	CCM@ZIF-8@HA@CS	Curcumin (CCM) was encapsulated into ZIF-8. Then, biocompatible polymers hyaluronic acid (HA) and chitosan (CS) were decorated on the surface of ZIF-8 by the layer-by-layer self-assembly technique	Under blue-light excitation, it presents a synergistic antibacterial effect	*E. coli* and * S. aureus*	107.1±9.5 nm	One-Step synthesis and LBL self-assembly technique	MIC=0.625μg/mL MIC=2.5μg/mL	(86)
Dual stimuli-responsive	RFP&o-NBA@ZIF-8	The RFP was loaded into the ZIF-8 and the o-NBA is modified into the porous structure of ZIF-8	The pH-responsive, light-triggered Zn(II) and antibiotic precise release for antibacterial therapy	*E. coli* and *S. aureus*	189.7±3.0 nm	One-pot reaction	≈100%	(87)
	Van/ZIF- 8/PDA	PDA coating was modified on the ZIF-8 loaded with vancomycin	NIR/pH dual stimuli-responsive platform (Van@ZIF-8@PDA) for synergistic photothermal/pharmacological antibacterial therapy	*E. coli* and *S. aureus*	170 nm	One-pot reaction	MIC=3.62 μg/mL	(88)
	Cur-ICG@ZIF-8/PLA/PCM	The Cur and ICG were co-encapsulated into the ZIF-8/polylactic acid. Then, Cur-ICG@ZIF-8/PLA was further coated with phase-change material	The combined action of curcumin and Zn(II) showed an excellent antibacterial effect. The curcumin and Zn(II) are released by the dual stimulatory response (NIR and pH)	*E. coli* and MRSA	1.10±0.30 μm	One-pot and vacuum freeze-dried	99.27%	(89)

MIC, minimum inhibitory concentration; MBC, minimum bactericidal concentration; NIR, near-IR; QDs, quantum dots.

Although pH-triggered structural collapse is considered a manner of controlled drug release, uncontrolled drug and Zn(II) leaks also plague researchers. Therefore, how to weaken the potential toxicity of the prematurely released Zn(II) and drugs to mammalian cells or normal organs is in the spotlight. To improve drug release behaviors and prevent the drug and Zn(II) leakage in the physiological environment, various biocompatible macromolecules have been modified on the surface of ZIF-8. Tu et al. (90) defined a unique Zn(II) releasing behavior to avoid the cytotoxicity of the prematurely released Zn(II) by constructing core-shell ZIF-8@PDA NPs. polydopamine (PDA) (91, 92), as a good biocompatible biopolymer, has a strong coordination effect on metal ions to limit the leakage of Zn(II). With the help of the bioadhesion of PDA coating to the bacterial surface, Zn(II) burst releasing was performed in a pH-dependent manner. Such a controlled Zn(II) releasing manner presented a promising prospect for antibacterial applications, but there is a distinct limitation, which is the lack of animal experimental support. A series of animal experiments need to be conducted to prove that this unique release behavior is feasible *in vivo*. Moreover, the targeting ability of ZIF-8 is still too weak to be applied. To achieve the precise target of pathogenic intracellular bacteria, hyaluronic acid (HA) as an anionic chain glycosaminoglycan (93) was reported to enhance targeting ability (94). Therefore, Zhang et al. (76) reported a pH-responsive MOF/antibiotic synergistic three-in-one nanocarrier tetracycline (Tet)@ZIF-8@HA (TZH). The ZIF-8 was functionalized by using the coordination between the carboxyl group of tetracycline to obtain TZH for the treatment of intracellular infections. In this work, the TZH is endocytosed by cells with the help of the HA-mediated pathway. Under acidic conditions, TZH releases Zn(II) and antibiotics, which synergistically inhibit intracellular bacterial infections.

**Figure 3:**
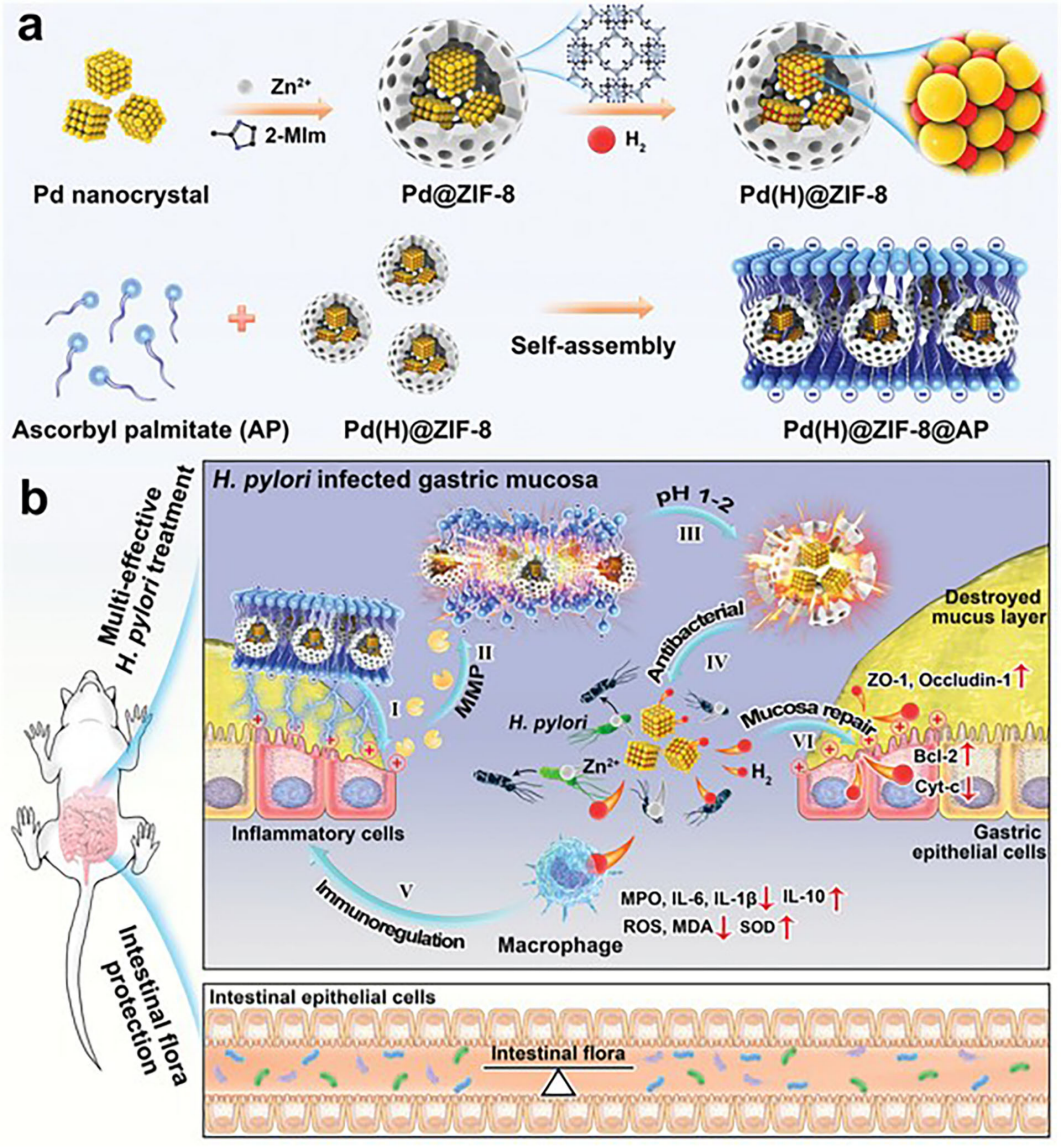
*Helicobacter pylori* treatment. **(A, B)** Schematic diagram showing the process of *H pylori* and restoring impaired gastric mucosa (75). Copyright 2021, WILEY-VCH.

Gastritis caused by *Helicobacter pylori* is a very serious problem that might lead to gastric cancer (95, 96). Due to the gastric acid microenvironment and the unique physiological state of the gastric inflammation site, Zhang et al. (75) developed a pH-responsive Zn(II) MOF hydrogen-generation system (Pd(H)@ZIF-8@AP). The Pd(H)@ZIF-8@AP was further coated by negatively charged ascorbyl palmitate (AP) hydrogel for targeting the *H. pylori* infection site (**Figure 3A**). The coating of Pd(H)@ZIF-8@AP endows inflammation-targeting ability by electrostatic adsorption with positively charged matrix metalloproteinase (MMP; IV collagenase) (97) (**Figure 3B**). Moreover, the AP ensures the release of Pd(H)@ZIF-8 *in situ* and prevents the leakage of Zn(II). The HA and AP coating endow the ZIF-8 with good biocompatibility and enhance targeting ability *in vivo*. They have great potential to be developed as effective, safe, and precise coatings for antibacterial applications.

**Figure 4:**
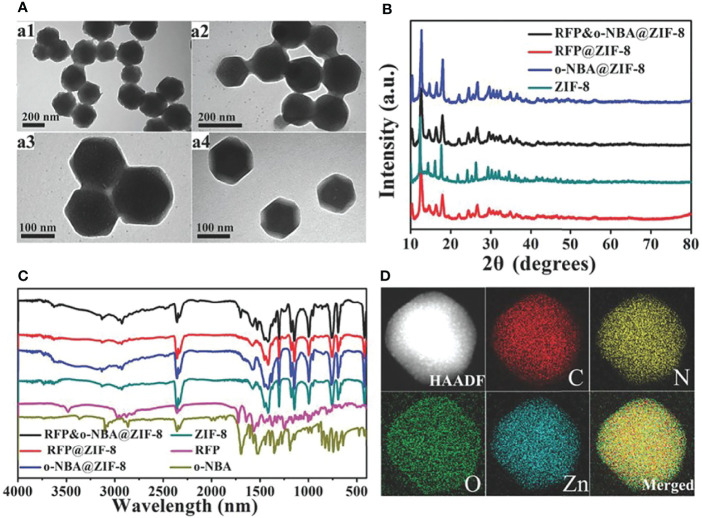
**(A)** Transmission electron microscopy (TEM) characterization. **(B)** X-ray diffraction (XRD). **(C)** Fourier transform IR (FT-IR). **(D)** (87). Copyright 2018, Wiley. Elemental mapping of RFP&o-NBA@ZIF-8.

In addition to pH-responsive ZIF-8, it is also designed as a light-triggered controllable nanoplatform. NIR as a trigger could collapse the nanostructure of ZIF-8 by photothermal effect and release Zn(II) to effectively kill bacteria. The light-triggered Zn(II) release manner has many advantages including high spatial resolution, fast effect, and controllability. This mode of release behavior has better specificity and is used for site-specific drug delivery. Yang et al. (83) designed a light-triggered ZIF-8-based chemical photothermal composite ICG@ZIF-8/PDA/Ag. The composite was constructed by encapsulating the photothermal agent (PTT) ICG inside and by decorating the surface with a PDA shell and Ag. Then, it could generate Ag ions *in situ* for a fast, long-term, and efficient antibacterial system. Local high temperature and metal ion release induced by NIR light cause damage to the bacteria. The antibacterial therapeutic effect was enhanced by the photothermal effect and the damage mediated by the release of excess Ag(I) and Zn(II). Wang et al. (98) proposed a combination therapy of Zn-doped MoS_2_ nanosheets and ZIF-8 (ZnDMZ) for photocatalytic sterilization. Under 660-nm light radiation, the ZnDMZ presented antibacterial efficacy of 99.9% against *Staphylococcus aureus* due to the synergy of photocatalytic effect, photothermal effect, and the released Zn(II). The pH-responsive and light-triggered ZIF-8 has been proved to improve drug release curves both *in vitro* and *in vivo* to varying degrees. To further improve Zn(II) release and enhance the therapeutic effects of Zn(II), dual stimulus-responsive nanodelivery systems (99) have been aggressively explored. The pH-responsive (75) and light-triggered ZIF-8 could accomplish enhanced drug release in spatial control and/or temporal control manner. It could respond to two physical stimuli to trigger drug release at the exact spot. It also provides many advantages including unprecedented control over drug delivery, sequential drug release manner, and excellent antibacterial potency. Song et al. (87) reported a pH-responsive and light-triggered MOF for antibacterial therapy, designated as o-NBA@ZIF-8 (**Figure 4**). The o-NBA@ZIF-8 is made up of “light-triggered” o-NBA (a pH-jump reagent), ZIF-8, and antibacterial agent rifampicin (RFP). The o-NBA could serve as a blocking cap to control the opening/closing of pore and a light-responsive functional component that produces acid *in situ* under the UV-light (365 nm) treatment. The acid produced by light stimulation leads to the degradation of ZIF-8 and the controlled release of antibiotics and Zn(II).

The sequentially responsive ZIF-8 showed a synergistic antibacterial effect against methicillin-resistant *S. aureus* (MRSA) attributed to the release of large amounts of Zn(II) and antibiotics. This cascade reaction triggers the release of Zn(II), which is innovative and provides a new perspective on the transformation of ZIF-8 nanosystems. Moreover, Su et al. (88) designed a NIR/pH dual stimulus-responsive nanoplatform Van@ZIF-8@PDA based on an imidazolate zeolite framework for synergistic photothermal/pharmacological antimicrobial therapy. In the article, Van@ZIF-8@PDA was modified with PDA to achieve high NIR photothermal conversion performance. The PDA coating improves its biocompatibility, dispersibility, and NP stability. The Van@ZIF-8@PDA released the encapsulated drugs through NIR triggering and pH-responsive manner, which could significantly reduce the off-target toxicity of drugs. The platform exhibited superior antibacterial ability and significantly reduced the survival rates of both *Mu50* and *Escherichia coli* through the combined effect of local photothermal damage, antibiotic membrane disruption, and Zn(II) overloading. As a result, the dual stimulus-responsive ZIF-8 nanoplatform provides a paradigm for optimizing the Zn(II) release curves *via* NIR triggering and pH-responsive mechanisms. Moreover, it is of high significance for enhancing the therapeutic efficacy and reducing side effects. This dual stimulus-responsive ZIF-8 nanoplatform as potential biomedicine with great biocidal property suggests great potential for treating drug-resistant bacterial infection.

As a widely used porous nanostructure, ZIF-8 has a homogeneous size and controllable pores (73, 74). Therefore, a variety of coatings could be modified to improve antibacterial efficiency. Different coatings endow ZIF-8 with different properties to overcome the challenges. With the development of polymer material science, the continuous innovation of these polymer coatings provides the possibility for the further development of ZIF-8. They could improve Zn(II) release curves, prevent the leak of Zn(II), and achieve the controlled release of Zn(II). However, the lack of active targeting remains an obstacle to its effective and precise drug delivery. For example, the collapse of ZIF-8 in the non-bacterial inflammatory sites would lead to the release of Zn(II) and drugs and severe off-target toxicity. In consequence, specifically targeting pathogens is necessary for improving therapeutic effectiveness. Next, we should focus on developing ZIF-8-based nanosystems with specific bacteria-targeting capabilities. Some materials that specifically target bacteria could be incorporated into ZIF-8 nanoplatform such as maltohexaose (100) and aptamer (101).

### Zinc Oxide and Zinc Oxide Nanocomposites

Bulk ZnO is recognized as a safe material (39) by the Food and Drug Administration (FDA) and could be biodegraded in the body (102). Nano-zinc oxide was explored by many researchers, as a form of zinc-based nanomaterials, and showed remarkable antibacterial activity in a variety of bacterial species (58, 103–105). Therefore, it is widely used for medical instruments (106), oral health products, the food industry, and other antibacterial applications (**Table 2**).

**Table 2:** Application of antibacterial ZnO-based and other zinc-loaded nanodelivery systems.

	Structural organization	Composition and modifications	Antibacterial mechanism and Zn release kinetics	Bacterial species	Size	Method	Test value	Ref
ZnO	ZnO NPs	Commercial zinc oxide powder	Photoactivated Zn(II) release	*Staphylococcus aureus*	50–70 nm	None	>95%	(107)
	NS-ZnO	ZnO nanoporous spheres	The rapid release of Zn(II) and the infiltration of ROS exert a synergistic antibacterial effect. The electrostatic forces promote the rapid release of Zn(II)	*S. aureus* and* Escherichia coli*	590 nm	Soft chemistry method	R ≈ 3.5	(108)
	ZnO	ZnO nanoflowers	Zn(II) release, photocatalysis, adsorption, or complexation synergistically produce a bactericidal effect	*S. aureus* and* E. coli*	38.31 nm	Low-temperature hydrothermal process	ZOI = 31.5 mm	(109)
	ZnO	ZnO nanopillars	Kills bacteria by cracking the surface and continuously releasing Zn(II) and free radicals without light irradiation	*E. coli*	1 μm	Solution growth method	96%	(110)
	Van @ZnO-PEP-MPA	MPA was covalently onto BSA-stabilized ZnO QDs	ZnO@BSA-MPA mediated the increased cell membrane permeation and increased antibiotic and Zn(II) influx	*S. aureus* and* Bacillus subtilis*	18 ± 8 nm	Solution growth technique and one-pot reaction	MIC = 2 μg/ml MIC = 1 μg/ml ≈100%	(111)
	ZnO/GO	ZnO nanoparticles (NPs), homogeneously anchored onto GO sheets	GO enabled the intimate contact of *E. coli* with ZnO NPs and Zn(II) as well and acted as the storage site for the dissolved Zn(II)	*E. coli*	4 nm	One-pot reaction	MIC = 1.7 μg ml^-1^	(112)
	ZnO@CQDs	The ZnO is decorated by carbon quantum dots (CQDs)	CQDs light-activated Zn(II) and ROS antibacterial activity	*S. aureus* and* E. coli*	19.92 nm	Sol–gel method	MIC = 6–8 μg/ml	(113)
	(PDA)-BP NSs/ZnO nanowires (NWs)	Titanium surface incorporated with ZnO NWs BP NSs and PDA	The photothermal effect enhances the Zn(II) release ability of ZnO, greatly improving antibacterial capability. NIR irradiation promotes the release of Zn(II)	*S. aureus* and* E. coli*	>200 nm	Liquid-phase exfoliation self-gravitation deposition of PDA	99.3%	(114)
	PLA MF-ZnO NPs	Sea urchin-like topography of ZnO nano-spicules	The ZnO NSs generate reactive oxygen species and allow the stabbing action of nano-spicules	*S. aureus* and* E. coli*	5 μm	Electrospray, electrospun, and interface self-assembly	N/A	(115)
	ZnO/PDA/RGDC	A hybrid ZnO/polydopamine (PDA)/arginine-glycine-aspartic acid-cysteine (RGDC) nanorod (NR) arrays are designed on the titanium (Ti) implants	Combination antibacterial derived from Zn(II), ROS, and physically punctured	*S. aureus* and *E. coli*	100 nm	Self-polymerization of dopamine and covalent immobilization of RGDC peptide	98.7% ± 0.1% and 99.9% ± 0.1%	(116)
	IL@ZnO	The surface ZnO was functionalized with an eco-friendly ionic liquid (IL)	IL@ZnO led to the spontaneous liberation of Zn(II) and the formation of ROS	*S. aureus* and* E. coli*	70 nm	Hydrothermal method and surface functionalization	MIC = 10 ± 2 μg/ml and MIC = 20 ± 2 μg/ml	(117)
	ZnO/TiO_2_ composites	ZnO/TiO_2_ hybrid structure	Light induced the release of Zn(II) from ZnO dissolution and ROS production. The release of Zn(II) tends to be saturated within 10 min under light conditions	*E. coli*	10–30 nm	Incipient wet impregnation method and calcination	99.9%	(118)
	DFT-C/ZnO hydrogels	Dopamine (DA) and folic acid (FA) cross-linked by Zn(II) coated around carbon quantum dot-decorated ZnO	The combination of antibacterial action was the released Zn(II) and the photothermal and photodynamic effects. Slow release of Zn(II) over 12 days	*S. aureus* and* E. coli*	Porous structure with a diameter of 50–60 µm	Polymerization process	99.99%	(119)
Other zinc-loaded nanomaterials	DexMa–PAAm nanogels	Dextran-cross-linked polyacrylamide nanogels were prepared, and Zn(II) was incorporated into the nanogels	The antibacterial activity was determined by the constant release of Zn(II). Slow release of Zn(II) within 24 h	MRSA	100 nm	Mini emulsion process	Delayed growth	(66)
	RC-TETA- PPIX-Zn	Protoporphyrin IX (PPIX) photosensitizer is covalently bound to epichlorohydrin/triethylenetetramine (TETA) for zinc chelation	The antibacterial activity was determined by the constant release of Zn(II). Light-triggered release of Zn(II)	*S. aureus* and* E. coli*	200–800 nm	Electrospinning and covalent bonding	99.99%	(120)

ROS, reactive oxygen species; ZOI, zone of inhibition; QDs, quantum dots; MIC, minimum inhibitory concentration; MRSA, methicillin-resistant Staphylococcus aureus.

The antibacterial mechanism of ZnO NPs involves direct contact with the cell wall, destroying bacterial cell integrity (107), the release of antibacterial abundant Zn(II), and ROS formation (108). Previous studies (58, 103) reported that ZnO NPs directly interact with bacterial membrane structure leading to the injury and rupture of the membrane (111). The increased bacterial membrane permeability mediated by ZnO NPs could facilitate the bacterial internal accumulation of Zn(II) and amplify the antibacterial effect of Zn(II) overloading therapy. Wang et al. (112) prepared ZnO/graphene oxide composites by a simple one-pot reaction using ZnO and graphene oxide (GO) and showed excellent antibacterial properties. The synergistic effect of GO and ZnO NPs resulted in unprecedented antibacterial activity of the composites. GO contributed to the dispersion of ZnO NPs, slowed down the dissolution of ZnO, and brought *E. coli* into close contact with ZnO NPs and Zn(II). The close contact enhanced the permeability of the bacterial membrane and the local Zn(II) concentration, thus leading to bacterial death. ZnO has demonstrated superior antibacterial properties by combination action of disrupting their membrane structure and leading to local Zn(II) burst.

Another huge advantage of ZnO is the synergistic effect of releasing large amounts of Zn(II) and producing ROS against bacterial infections (39). For ZnO, large numbers of valence band holes and/or conduction band electrons were thought to be available to trigger redox reactions and ROS generation in cellular environments (121, 122). With the production of ROS, Zn(II) is also released from ZnO NPs. Lucas-Gil et al. (108) prepared a ZnO cluster with a porous spherical structure by a soft chemical method. The ZnO clusters exhibited higher antibacterial and antifungal activities and lower cytotoxicity as compared to commercial ZnO NPs. Chai et al. (123) developed a hybrid NP-containing silica nanorattles (SNs) and ZnO, which could inhibit the growth of MRSA. SNs could gather ZnO NPs to enhance the production of free radicals and Zn(II) owing to the mesoporous nanomaterials, thus shortening the effective touching distance between free radicals and MRSA. The ZnO@SN NPs exhibit a higher inhibition efficiency than free ZnO NPs and SNs against MRSA *in vitro* and *in vivo*. Quantum dots (QDs) have been a hot topic since they have been discovered. Because of their water solubility, photostability, and easy functionalization, they have aroused great interest in various fields. Moussa et al. (124) reported that ZnO@APTMS/Cu QDs generate high amounts of H_2_O_2_ •OH and O_2_^•−^ radicals. The production of Zn(II) and ROS gives the ZnO@APTMS/Cu QDs multimode antibacterial activity.

**Figure 5:**
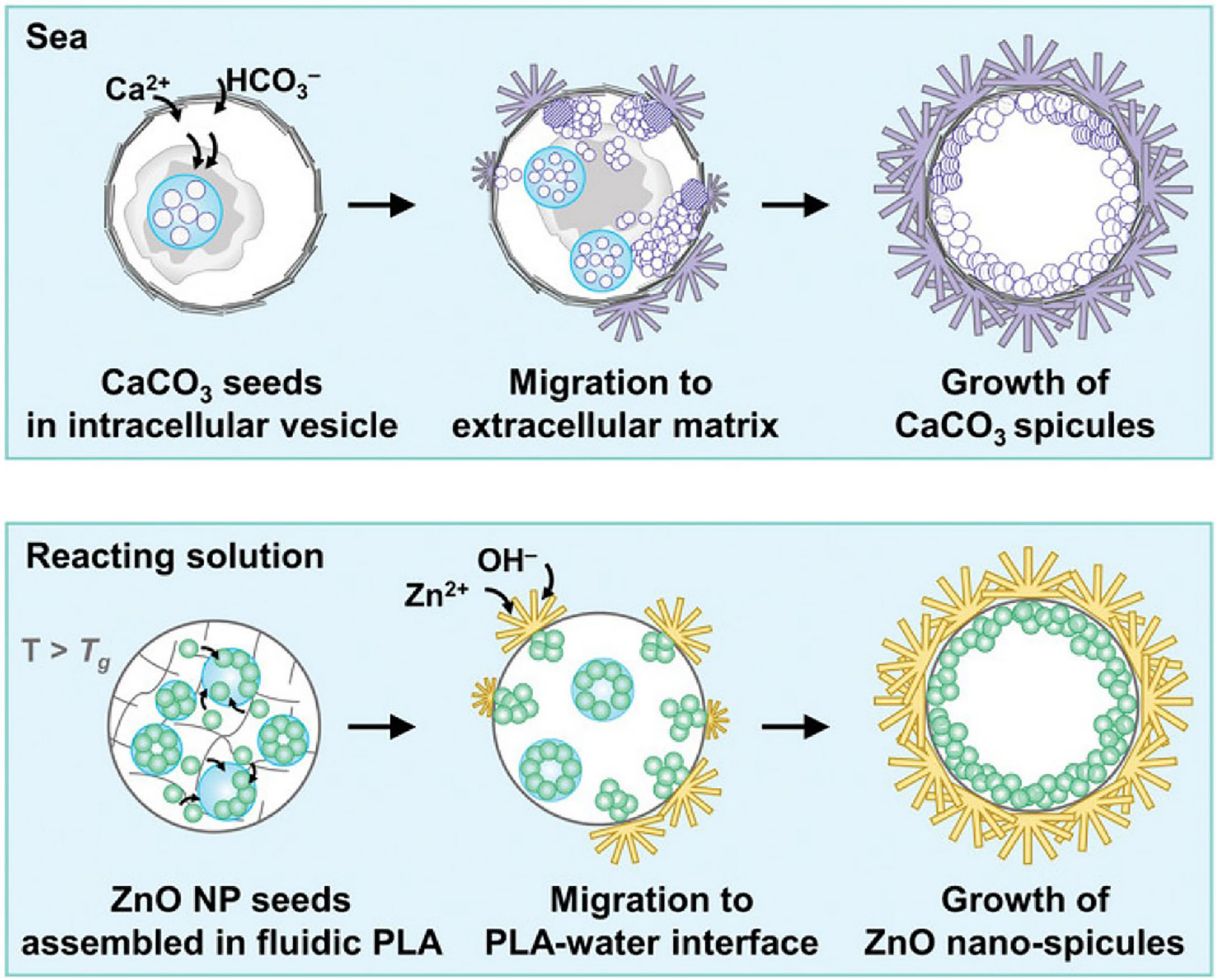
The formation process of artificial micro-urchin with ZnO nano-spicules (115). Copyright 2021, Wiley.

In addition to ZnO NPs, a variety of ZnO-based nanomaterials have been explored and developed for antimicrobial therapy. The sophisticated management of nanomaterials is essential for the efficiency of the antibacterial activities of ZnO. In recent studies, researchers designed various ZnO nanomaterials by morphological modulation including ZnO nanoflowers (109), nanowires (NWs) (114), nanorod (106, 116), nanopillar (110), and nanoneedles (115). Interestingly, Kim et al. (115), inspired by the sea urchin acicular structure, reported the super-hydrophilic and bactericidal surface of ZnO nanoneedles grown from micron-sized polylactic acid (PLA) beads and fibers (**Figure 5**). In this paper, the authors mainly focused on the physical behavior of sea urchin-like zinc oxide. There are a few contents for exploring the antibacterial mechanism of Zn(II). Future efforts will focus on biomedical applications. Bains et al. (117) prepared IL@ZnO nanosheets *via* a hydrothermal method and further functionalized them with an eco-friendly ionic liquid (IL). The IL@ZnO exhibits excellent antibacterial activity against *E. coli* and *S. aureus*. More importantly, the authors demonstrated that IL@ZnO material could serve as a potential antimicrobial coating surface for cotton fabric by fabricating IL@ZnO onto the surface of cotton fabric. Therefore, it might be utilized as a biomaterial for biomedical applications, especially in medical masks, to reduce the risk of the spread of infectious diseases. Despite showing a superior antibacterial performance, the lack of safety evaluation *in vivo* makes ZnO with different morphologies difficult in clinical practice. Researchers should focus on exploring the metabolic kinetics distribution of ZnO with different morphologies *in vivo* to lay a foundation for further application.

**Figure 6:**
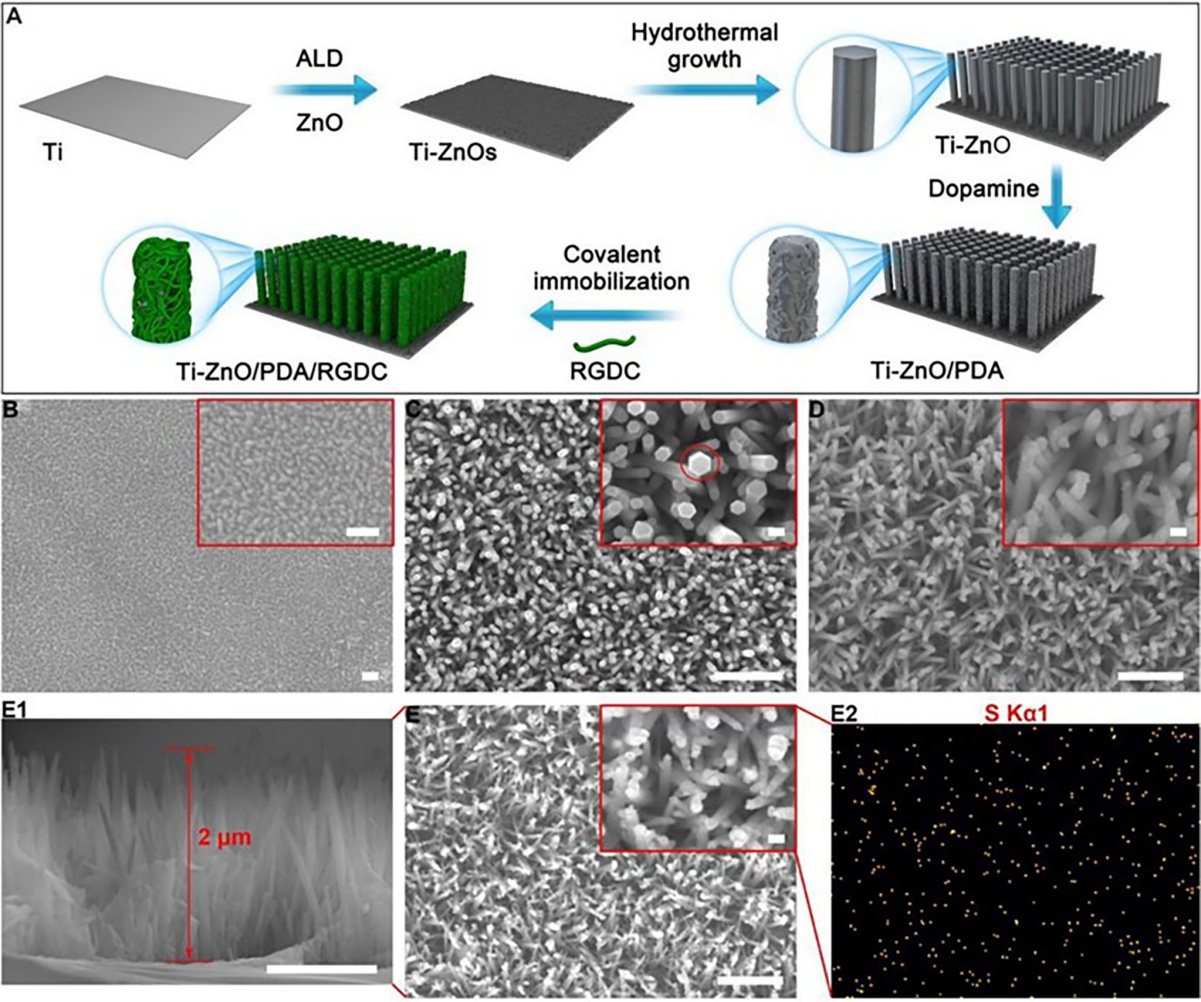
**(A)** Schematic illustration of the fabrication process of the hybrid ZnO/PDA/RGDC NR arrays. Scanning electron microscopy (SEM) images of **(B)** Ti-ZnOs (scale bars = 100 nm), **(C)** Ti-ZnO, and **(D)**Ti-ZnO/PDA (116). Copyright ^©^ 2017 American Chemical Society. **(E)** SEM images of Ti-ZnO/PDA/RGDC; (E1) cross-sectional image of Ti-ZnO/PDA/ RGDC; (E2) Elemental mapping of Ti-ZnO/PDA/RGDC [scale bars = 100 nm (inset figures = 1 μm)].

Although ZnO NPs and ZnO-based nanomaterials demonstrate excellent multi-mode antibacterial properties, ZnO NPs and ZnO-based nanomaterials as metal-based NPs inevitably produce cytotoxicity and serious adverse reactions. How to avoid the toxicity of ZnO-based nanomaterials is an important problem. Fang et al. (114) designed a photothermal antimicrobial platform that combines PDA-BP NSs/ZnO NWs on the titanium (Ti) matrix of the implant to eliminate biofilm-related infections. In this platform, PDA is modified on the implant to form a surface-adhesive nanocoating. With the synergistic effect of BP NSs and PDA, the PTT antimicrobial ability was significantly enhanced and made bacteria more sensitive to Zn(II) undergoing the 808-nm irradiation. The photothermal effect also amplified Zn(II)-overloading therapy against *S. aureus* and *E. coli* infection. The NIR synergistic antimicrobial strategy has good biosafety and can effectively control bacterial infection without damaging normal tissues. Li et al. (116) reported the hybrid ZnO/PDA/arginine-glycine-aspartic acid-cysteine (RGDC) nanorod (ZnO/PDA/RGDC) arrays on titanium (Ti) implants (**Figure 6**). The coating of titanium (Ti) implants was composed of ZnO/PDA/RGDC nanorods and had superior antibacterial properties and enhanced the osteoinductivity ascribed to the bio-functionalization of ZnO/PDA/RGDC nanorods. PDA, as a stabilization agent in the fabrication of diverse organic−inorganic materials with specific functionalities, has an excellent ability to manage the release of Zn(II) and balance antibacterial activity and cytotoxicity. The PDA coating endows the nanostructure with good biocompatibility, reducing the toxicity and enhancing the antibacterial performance of Zn(II).

ZnO NPs and ZnO-based nanomaterials exhibit strong antibacterial effects even when administered in small amounts. However, the antibacterial activity of zinc oxide is affected by many factors. Firstly, the antibacterial efficiency of ZnO NPs increased with the decreasing particle size (125). ZnO NPs exhibit enhanced antibacterial properties than bulk ZnO with micron-scale at the same concentration. The smaller-size ZnO NPs showed better antibacterial activity. The enhanced bactericidal efficacy of ZnO NPs attributes to a higher specific surface area. The smaller ZnO NPs could release more Zn(II) and ROS and result in a better antibacterial effect.

Various morphologies of ZnO also significantly impact the toxicity of ZnO to bacteria. The flower-shaped ZnO has revealed higher biocidal activity against *S. aureus* and *E. coli* than the spherical and rod-shaped ZnO (126). Such shape-dependent antibacterial activity behavior was explained that ZnO nanostructure with different morphologies has different active facets, which may lead to improved antibacterial activity (63). Therefore, these features that affect the antibacterial performance of ZnO nanostructure by the size and shape of ZnO provide us with ideas to design smaller, multi-active surface oxidizing nanomaterials.

Bulk ZnO particles are generally considered safe by the FDA and are widely used in food additives, cosmetics, and other fields (63, 125). However, toxicity occurs when the size of ZnO is reduced to the nanostructure. ZnO NPs are toxic to many mammalian cells, such as human bronchial epithelial cells, human kidney cells, and human alveolar adenocarcinoma cells (127–129). Therefore, the ZnO NPs are limited to ointment, medical dressing, and other forms to treat local skin infections. To further expand the antibacterial application of ZnO nanomaterials, the toxicological effect of ZnO nanomaterials should be elucidated. Moreover, some biomimetic or engineered biomimetic coatings like erythrocyte membrane (130, 131) and platelet (132) for ZnO nanomaterials could be applied to improve the biosafety of ZnO nanomaterials.

### Other Zinc-Based Nanostructure

Nanogels could form a dense network through intermolecular cross-linking (65), which is an ideal delivery material for metal ions or small molecule drugs (133). However, the major limitation of nanogels or hydrogels is uncontrollable drug release. In order to achieve the controllable release of Zn(II), Malzahn et al. (66) reported enclosed nanogels based on diisocyanate chemistry reaction for the shell-forming. The enclosed nanogels could control the release pattern and reduce the Zn(II) release rate. In this paper, a dextran (DexMa)-polyacrylamide (PAAm) nanogel was prepared by a microemulsion method. Then, the multiple methacrylate portions of the dextran side chain would react with PAAm and thus act as a cross-linking agent to form the shell of the nanogel. This cross-linked shell limits water penetration and impedes the expansion of the nanogels, thus increasing the retention of Zn(II) and prolonging the release of Zn(II) for achieving sustained antibacterial action. The nanogels were modified to retain the Zn(II) over a longer period and be better suited for biomedical applications. This strategy of preparing nanogel with shell solves the problem of uncontrollable behavior of Zn(II) and achieves slow and long-term release of Zn(II). More important, it provides a promising treatment for repeated long-term infection of wounds, especially for diabetics. However, in this article, the nanogels only demonstrate the antibacterial effect of Zn(II), Zn(II) release behaviors, and kinetics at the theoretical level. Further research is needed on pharmacodynamics and animal models. Moreover, Dong et al. (120) have developed a nanofiber with the controllable release of Zn(II). In this paper, cellulose acetate was used to produce nanofibers by electrostatic spinning. Proto-porphyrin IX (PPIX) photosensitizer was fixed to the surface of the nanofibers by covalent grafting, and then the RC-TeTA-PPIX-Zn was obtained by conjugate chelating Zn(II) with porphyrin ring. Under xenon lamp irradiation, photosensitizer produces singlet oxygen (^1^O_2_) primary bactericide and releases Zn(II). The presence of the Zn(II) enhanced intersystem crossing, leading to higher singlet oxygen yield and improving the bactericidal efficiency. However, this light-responsive controlled release behavior of Zn(II) is severely limited to clinical translation owing to xenon lamp radiation. The researchers need to further optimize the nanosystems for practicality and safety.

For zinc ion overloading therapy, different zinc-based nanodelivery systems offer different advantages depending on their composition and structure. Compared with ZnO nanomaterial, ZIF-8 has many advantages. I) ZIF-8 has a homogeneous size and controllable pores. The controllable pores and high porosity allow ZIF-8 to act as an ideal nanocarrier for drug delivery. ZIF-8 could encapsulate a large number of antibiotics and zinc ions to play a synergistic antibacterial effect. II) ZIF-8 could respond to the collapse of nanomaterials caused by low pH. This pH-responsive feature of ZIF-8 allows the controlled release of Zn(II) at the infection site and reduces off-target toxicity. III) ZIF-8 is safer than ZnO nanomaterials. The ZnO could accumulate on the surface of the cell membrane and induce cell damage due to the positively charged Zn^2+^. IV) ZIF-8 has been extensively studied and developed. Recently, it was developed to deliver drugs, microRNA, and gas to enhance the antibacterial effect. ZIF-8, which is loaded with gas or microRNA, is highly effective against bacteria in combination with zinc ion overloading therapy. However, the antibacterial mechanism of ZnO nanomaterial is involved in Zn(II) release, membrane dysfunction, generation of ROS, etc. The combination of multiple antimicrobial modes makes the ZnO nanomaterial more lethal. Nanogels have been stranded out in recent years as innovative nanodelivery systems for overcoming the limitations of conventional nanodelivery (134). Compared with ZnO nanomaterial and ZIF-8, the advantages of nanogels include lower drug leakage, biocompatibility, and degradability (135). The nanogels formed by cross-linking of polysaccharides have been demonstrated to have greater safety for *in vivo* applications. Moreover, as an antibacterial Zn(II) carrier for Zn(II), it is rarely explored. This means that the antibacterial therapy of nanogels loaded with Zn(II) has broad prospects. The future goal of the usage and development of nanogels loaded with Zn(II) would be with robust biocompatible features and programmed responses. Moreover, inspired by nanosponge, nanogels formed by cross-linking molecules, peptides, and polymers with strong metal ion chelating abilities also show promising prospects for the application of the Zn(II) deprivation strategy.

### Zn(II) Deprivation Strategy

Zn(II) deprivation strategy is used to break the bacterial intracellular Zn(II) homeostasis through the action of Zn(II) chelators. When the bacterial intracellular Zn(II) homeostasis is out of balance, it would seriously interfere with the normal physiological and metabolic processes of bacteria. Therefore, Zn(II) deprivation strategy has emerged as a promising approach to effectively kill bacteria or reverse antibiotic resistance. Zn(II) chelation agents, such as *N*,*N*,*N*′,*N*′-tetraacetate (EDTA) (136), AMA (29, 137), and other small molecules (138, 139), are powerful tools to chelate Zn(II) in the bacterial infection microenvironment and cause Zn(II) metabolism disorder to achieve dysregulation of related functional proteins and cause bacterial death. However, those Zn(II) chelation agents also face several challenges. Here, we will discuss the applications of the Zn(II) deprivation strategy.

### Zn(II) Deprivation Nanosystem

**Figure 7:**
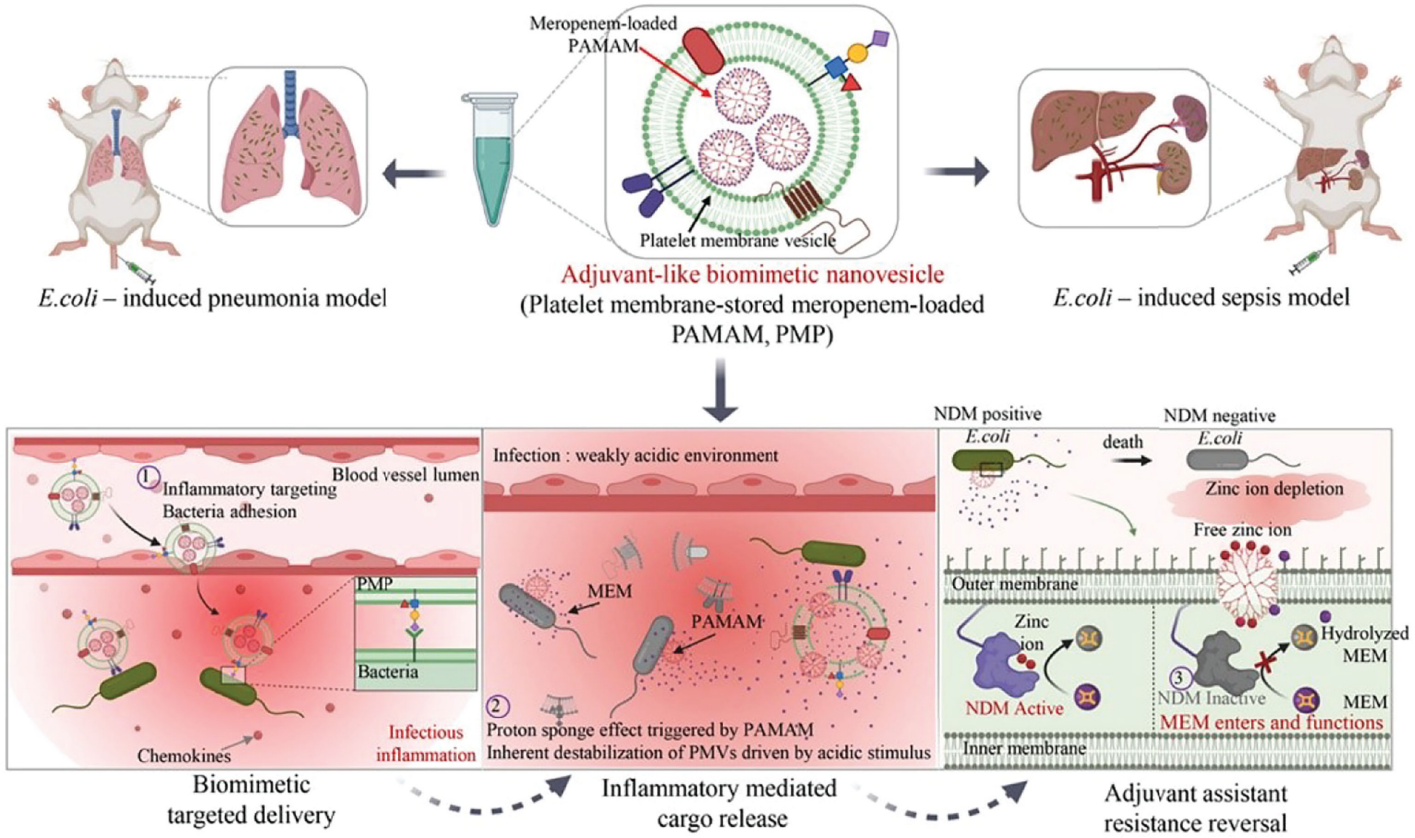
Schematic illustration of adjuvant-like biomimetic nanovesicles could target the bacterial infection microenvironment and reverse carbapenem resistance for NDM-producing bacterial infections (142). Copyright 2021, Elsevier B.V.

Due to the lack of pathogen targeting, small-molecule Zn(II) chelation would result in indiscriminate chelation of Zn(II) in mammals, which impairs the biological functions of normal cells. Moreover, as a dense and strong barrier, the outer membrane of Gram-negative bacteria prevents many drugs from entering the bacteria (140, 141). How to break through the outer membrane barrier is also a very challenging problem. Wu et al. (142) first found that polyamidoamine (PAMAM) could efficiently break through the outer membrane barrier and reverse bacterial resistance through Zn(II) deprivation strategy. As a metal β-lactamase with Zn(II) as the active site (31, 143), NDM can hydrolyze almost all clinically available antibiotics and be a key factor in antibiotic resistance. There is an urgent need to find ways to overcome NDM-induced drug resistance. Therefore, they constructed a platelet membrane nanovesicle containing PAMAM and meropenem. The nanovesicle could target bacterial infection sites and adhere to the surface of the pathogen *via* the inflammatory targeting properties of platelets (**Figure 7**). Owing to the acid environment of bacterial infection, the amino groups of PAMAM are protonated and present a strong positive charge density, which mediates the formation of nanoscale pores in platelet membrane nanovesicle and bacterial outer membrane. In the bacteria periplasm, PAMAM inhibits NDM enzyme activity by competitively chelating Zn(II) of NDM active sites, thus preventing meropenem from hydrolysis. It provides a new angle for the treatment of NDM-producing bacterial infections. This nanovesicle solves two challenges: targeting and breaking through the outer membrane barrier. More important, this Zn(II) deprivation nanovesicle effectively inactivates NDM-1, reverses the antibiotic resistance, and revives meropenem. The nanovesicle offers hope for the treatment of patients with clinical infection NDM-1 and presents a great possibility for clinical translation.

### Other Deprivation Strategies

Macromolecular substances like calprotectin (an antimicrobial protein released by neutrophils) (144) and antimicrobial peptides (145) also perform an excellent ability to chelate Zn(II). Ma et al. (145) developed an antimicrobial peptide named thanatin, which could break through the bacterial outer membrane barrier and competitively displace the Zn(II) on NDM-1. The results suggest that thanatin has the property to damage the bacterial outer membrane and restore the susceptibility of NDM-1-producing pathogens to β-lactam antibiotics by depriving the NDM-1 enzyme active site of Zn(II). The unique antibacterial mechanism of thanatin provides a strategy for combating infection by NDM-1 pathogens. Some studies (144, 146) found that calprotectin has broad-spectrum antimicrobial activity against many microorganisms including bacteria and fungi pathogens. The antibacterial mechanism of calprotectin (147) involves competition with many transition metal elements to limit the availability of essential metal nutrients such as Ca(II), Zn(II), and Cu(II). Moreover, although various hydrogels (148, 149) and microparticles (150) have been used to chelate Zn(II) in the microenvironment, they were rarely used in the field of antibacterial infection. Sonamuthu et al. (151) reported an MMP-9-responsive dipeptide-tempted natural protein hydrogel for infected diabetic wound therapy. The author explored a facile multifunctional silk protein hydrogel matrix encapsulating bioactive curcumin and l-carnosine dipeptide. The l-carnosine dipeptide inactivated the MMP-9 *via* its strong chelation action of Zn(II) from the MMP-9 active center. The curcumin biomolecules showed energetic antimicrobial efficacy, wound healing, and tissue regeneration ability. The l-car@cur/SF hydrogel matrix could be an effective diabetic wound dressing material to reinstate the functions of the chronic wound site.

Compared with the discovery of novel antibiotics, the development of the zinc ions deprivation strategy to reverse antibiotic resistance is considered to be the most cost-effective option against MDR bacteria. Although the zinc ion deprivation strategy shows promising prospects, certain challenges still limit its clinical translation and commercial application. Zn(II) chelators always indiscriminately deprive Zn(II) of bacterial and mammalian cells *in vivo*, resulting in multiple impaired biological functions and high off-target toxicity. Moreover, due to the influence of the complex bacterial infection microenvironment, the protonation characteristics of the PAMAM mentioned above are interfered with, thus significantly reducing its Zn(II) deprivation efficiency and specificity. How to effectively and accurately chelate Zn(II) in bacteria is the key to fighting drug-resistant bacteria *in vivo* and pushing Zn(II) chelators to clinical application.

## Conclusions and Perspectives

In this paper, we review the applications of Zn(II) interference therapy in bacterial infection. Zn(II) is one of the most important transition elements in the metabolic activities of living organisms. Zn(II) homeostasis plays a decisive role in bacterial colonization, proliferation, and even the occurrence, development, and spread of antibiotic resistance. Therefore, Zn(II) deprivation could effectively inactivate the enzymes that perform important physiological functions and result in bacterial metabolic dysfunction or failure of the bacterial resistance mechanism. For Zn(II) overloading strategy, the excess Zn(II) could also cause different forms of damage to bacteria, such as oxidative stress, and protein dysfunction. Given the antibacterial mechanisms, Zn(II) interference therapy could avoid the antibiotic resistance mechanism or reverse the antibiotic resistance and provide a powerful weapon to kill MDR bacteria. The Zn(II) interference therapy with the help of bioactive nanomaterials is expected to amplify the effect of Zn(II) therapy and overcome antibiotic resistance. Zn(II) interference therapy provides an alternative means for the treatment of MDR bacterial infections.

For Zn(II) overloading strategy, as a zinc-centered MOF, ZIF-8 has been developed as an antibacterial delivery system due to its unique acid response mechanism and porous structure. Although it exhibits good antibacterial action, many organic ligands produced by dissociation of ZIF-8 show inherent toxicity and low biocompatibility, limiting their widespread application. Therefore, biosafe MOF should be explored. Some biosafe ligands and nucleobases such as amino acids should be developed as novel ligands of MOF. Moreover, various forms of ZnO and complexes have been also developed for antibacterial applications for Zn(II) overloading strategy. The antibacterial mechanism of ZnO involves the production of intracellular ROS, the release of Zn(II), and mechanical damage to bacterial membranes. The antibacterial activity of ZnO NPs is affected by many factors including particle size, concentration, morphology, surface modification, and other factors. However, the toxicity of ZnO NPs to mammalian cells should not be ignored, and more data are needed to determine long-term health risks. The long-term biosafety, biodistribution, and toxicity of various morphologies of ZnO nanomaterials remain to be further studied. To further expand the antibacterial application of ZnO nanomaterials, some biomimetic and engineered biomimetic coatings could be modified to ZnO nanomaterials. Moreover, hybrid ZnO nanomaterials have been proven to possess improved antimicrobial activity for synergistic effects. ZnO nanomaterials can be combined with enzymes or photosensitizers for reducing the dosage of ZnO.

For Zn(II) deprivation strategy, Zn(II) is an important transition element that both the human body and pathogens depend heavily on. How to deprive Zn(II) of bacteria more precisely is a very meaningful scientific problem. Fortunately, due to the demand for Zn(II) in the human body, there are measures to compete for Zn(II) in the human immune system. For example, calprotectin, as an antimicrobial protein released by neutrophils, could inhibit bacterial growth by limiting Zn(II) availability. It could be modified and designed into NPs that deliver antibiotics while chelating zinc ions. Moreover, improving the effectiveness of host-mediated Zn(II) restriction at the pathogen–host interface and reducing the acquisition of zinc by bacteria to limit the growth of bacteria are also promising solutions. In addition, some metal ions, such as Pb^2+^, Ni^2+^, and Bi^3+^, could act as “zinc traps” to replace zinc at active sites on functional proteins and embed themselves into the Zn(II)-utilization pathway and further damage the downstream Zn(II)-related metabolism state inside bacteria. In conclusion, Zn(II) interference therapy, based on nanodelivery systems, carves a new path for combating drug-resistant bacterial infections, benefiting from unique mechanisms, inherent targeting properties, and high therapeutic efficiency. With the development of advanced nanomaterials, nanomaterial-based zinc ion interference therapy will provide significant contributions to fighting MDR bacterial infection, and the clinical translation of Zn(II) interference therapy will be achieved in the near future.

## Author Contributions

YW: writing—original draft preparation. JW: writing—original draft preparation. SW: writing—original draft preparation. RZ: writing—original draft preparation. KZ: writing—review and editing. ZZ: writing—review and editing. JL: writing—review and editing. SQ: writing—review. JS: writing—review. All authors listed have made a substantial, direct, and intellectual contribution to the work and approved it for publication.

## Funding

This work was supported by grants from the National Natural Science Foundation of China (Nos. 82172762, 31900991, 82073395, 82073787, U2004197, 22122409), Innovation Talent Support Program of Henan Province (No. 21HASTIT043), Postdoctoral Science Foundation of China (No. 2020TQ0288, 2021M690140), and Youth Talent Promotion Foundation of Henan Province (No. 2021HYTP047), Outstanding Youth Foundation of Henan Province (222300420020).

## Conflict of Interest

The authors declare that the research was conducted in the absence of any commercial or financial relationships that could be construed as a potential conflict of interest.

## Publisher’s Note

All claims expressed in this article are solely those of the authors and do not necessarily represent those of their affiliated organizations, or those of the publisher, the editors and the reviewers. Any product that may be evaluated in this article, or claim that may be made by its manufacturer, is not guaranteed or endorsed by the publisher.
